# PONV management in patients with QTc prolongation on the EKG

**DOI:** 10.3389/fphar.2020.565704

**Published:** 2021-01-12

**Authors:** S. Soghomonyan, N. Stoicea, W. Ackermann, S. P. Bhandary

**Affiliations:** Department of Anesthesiology, The Ohio State University Wexner Medical Center, Columbus, OH, United States

**Keywords:** PONV, postoperative nausea and vomiting, QTc, anesthesia, perioperative period, torsade de pointes

## Abstract

Postoperative nausea and vomiting (PONV) is a commonly encountered problem in surgical practice. It delays discharge from the post-anesthesia care unit, requires additional resources to treat, and may increase the morbidity in some patients. Many effective drugs are available to treat or prevent PONV, however many of these drugs have the potential to prolong the QTc on the electrocardiogram (EKG) and increase the risk of serious ventricular arrhythmias, in particular, torsade de pointes. The QTc prolongation may be a manifestation of a genetic mutation resulting in abnormal myocyte repolarization or it may be acquired and associated with the use of various medications, electrolyte disorders, and physiological conditions. Patients predisposed to QTc prolongation presenting for surgery constitute a challenging group, since many drugs commonly used for PONV management will put them at risk for perioperative serious arrhythmias. This is an important topic, and our mini-review is an attempt to highlight the problem, summarize the existing experience, and generate recommendations for safe management of PONV for patients, who are at increased risk of QTc prolongation and arrhythmias. Focused prospective studies will help to find definitive answers to the discussed problems and challenges and develop specific guidelines for clinical application.

## Introduction

Postoperative nausea and vomiting (PONV) is a common phenomenon complicating the postoperative course which may result in significant discomfort to the patient, delay the discharge, and increase the cost of treatment (Gan et al., 2020). Various drugs have been successfully used to prevent and treat PONV (Table 1). Many of these drugs share the potential to prolong the QTc on EKG, something that increases the risk for development of ventricular arrhythmias, specifically, Torsade de Pointes (TdP): a phenomenon first described in 1966 by Dessertenne (Charbit et al., 2005; Curigliano et al., 2009; Drew et al., 2010; Singh et al., 2018; Berul, 2020) Indeed, most of the episodes of QTc prolongation seen with the use of antiemetic drugs remain clinically silent, self-limited, and rarely require active intervention. Nevertheless, there is a group of patients with hereditary or acquired risk factors for development of long QTc and TdP, and these patients require special attention during the perioperative period. EKG monitoring and an action plan to promptly treat TdP and other arrhythmias, should they occur, is mandatory.

**TABLE 1:** Drugs commonly used to prevent and treat PONV (Charbit et al., 2005; Curigliano et al., 2009; Marbury et al., 2009; Owczuk et al., 2009; Drew et al., 2010; Brygger and Herrstedt, 2014; Chu et al., 2014; Perkins et al., 2015; Tracz and Owczuk, 2015; Täubel et al., 2017; Hellström and Al-Saffar, 2018; Lai and Huang, 2018; Hansen, 2019; Staudt and Watkins, 2019; Berul, 2020; Gan et al., 2020).

Drug	Mechanism of action	Doses to treat/Prevent PONV	Timing	QTc prolongation	Adverse effects	Comments
Ondansetron	5-HT3-RA	4–8 mg	Near the end of surgery	Mild temporary QTc prolongation	Headache, fatigue, constipation or diarrhea, drowsiness, fever	Safe for PONV prophylaxis when used in recommended doses
Granisetron	5-HT3-RA	0.35–3 mg IV	Near the end of surgery	QTc prolongation less pronounced compared to ondansetron	Headache, constipation or diarrhea, weakness, fever, dizziness	Safe for PONV prophylaxis when used in recommended doses
Palonosetron	5-HT3-RA	0.075 mg IV	At induction	No significant effects on QTc	Headache, constipation, diarrhea, dizziness	No EKG studies were done during the time of cmax
Metoclopramide	D2RA, 5-HT3 RA, 5-HT4 RA	10–20 mg I.V.	Near the end of surgery	Moderate risk for QTc prolongation	Agitation, insomnia, restlessness, akathisia, extrapyramidal motor symptoms	Doses >20 mg are rarely administered to avoid the side effects. However, lower doses have a weaker antiemetic effect compared to other drugs
Amisulpride	D2RA, D3RA	5 mg	At induction	No significant effects on QTc	Mild increase in prolactin level	Doses used for PONV prophylaxis do not significantly prolong the QTc
Droperidol	D2RA	<= 1.25 mg I.V.	At induction or near the end of surgery	Mild QTc prolongation	Drowsiness, restlessness, hyperactivity, anxiety	Safe for PONV prophylaxis when used in recommended doses
Haloperidol	D2RA	0.5–1 mg I.V.	At induction or near the end of surgery	Mild QTc prolongation	Drowsiness, restlessness, hyperactivity, anxiety	Safe for PONV prophylaxis when used in recommended doses
Midazolam	Benzodiazepine receptors	1–2 mg I.V.	At induction of anesthesia	No significant effects on QTc	Sedation, amnesia	May not be appropriate for older patients
Ephedrine	Predominantly indirect adrenergic stimulant, α1 β1, and D1 direct agonist	0.5 mg/kg I.M.	Near the end of surgery	Mild QTc prolongation	Hypertension, tachycardia	May need precautions in patients with LQTS, who are sensitive to sympathetic stimulation
Dexamethasone	Glucocorticoid receptor agonist, possibly, neurokinine receptor antagonist, anti-inflammatory action	4–8 mg I.V.	At induction	No action of QTc interval	Potential risk of infections, hyperglycemia	Effective for prophylaxis, not treatment of PONV.
Promethazine	H1A, D2RA2, α1RA, NMDARA, MChRA	6.25–12.5 mg I.V.	May be used as a rescue drug in PACU.	Moderate risk of QTc prolongation	Sedation, dizziness, double vision, dry mouth	Lack of influence on transmural dispersion of repolarisation makes the risk of TdP very low. IV and IM, oral, and rectal administration is safe. SC injection may cause tissue damage
Aprepitant	NK1RA	40–80 mg P.O.	Preoperative	No action of QTc interval	Fatigue, diarrhea, dizziness, hiccups	More effective in reducing the incidence of postoperative vomiting rather than nausea
Fosaprepitant	NK1RA	150 mg I.V.	At induction. Infuse over 30 min	No action of QTc interval	Constipation, diarrhea, headache, neutropenia	
Propofol	Decrease in the rate of GABA dissociation from the GABAA receptor, possible 5-HT3-RA1 and D2RA2	20 mcg/kg/min I.V.	Throughout surgery	No action of QTc interval	Sedation, respiratory depression	Short acting effect. May be used as a rescue medication
		20 mg bolus I.V.	End of surgery, PACU	No action of QTc interval		
Scopolamine	MChRA	1.5 mg transdermal patch	Prior evening or 2 h before surgery	No action of QTc interval	Dry mouth, Visual disturbance, dizziness	Central and peripheral anticholonergic effects may limit the use in elderly
Gabapentin	Inhibition of voltage-gated calcium channels; GABAB receptor activation	600–800 mg P.O.	Preoperative	No action of QTc interval	Drowsiness, dizziness, drowsiness, blurred vision	The therapeutic effect against PONV is masked, when propofol is used during anesthesia

5-HT3-RA, 5-HT3 receptor antagonist; D2RA, Dopaminergic type 2 receptor antagonist; D3RA, Dopaminergic type 3 receptor antagonist; 5-HT4RA, 5-HT4 receptor agonist; H1A, histamine type 1 receptor antagonist; NK1RA, neurokinin 1 receptor antagonist; α1RA, alpha 1 receptor antagonist; NMDA RA, NMDA receptor antagonist; MChRA, M cholinergic receptor antagonist; QTc, QT corrected; PONV, postoperative nausea and vomiting; LQTS, long QT syndrome; TdP, Torsades de pointes; GABA, Gamma Aminobutyric Acid; GABAA, GABA A receptor; GABAB, GABA B receptor.

## Prolonged QTc and Anesthesia

Abnormal cardiac repolarization is a common occurrence and is identified as prolonged QTc interval. QTc prolongation can be inherited, acquired or both. A significant number of patients present with QTc prolongation prior to anesthesia and surgery, even without any use of drugs with QTc prolonging properties, including antiemetic medications. It is well known that many patients who have a genetic predisposition to the long QT syndromes (LQTS) never develop a QTc prolongation, and additional contributing factors are required, including medications, to trigger ventricular arrhythmias (Curigliano et al., 2009; Drew et al., 2010). Nevertheless, these patients are considered a group of high risk, since their risk for sudden cardiac death is increased more than twofold and is even higher among younger patients (Brygger and Herrstedt, 2014). According to Staudt and Watkins. (2019), the estimated incidence of hereditary QTc prolongation among newborns is 1 in 2,500 (Staudt and Watkins, 2019).

Thus, despite the low chance of developing TdP or other life-threatening arrhythmias, patients with a preexisting QTc prolongation need proper anesthesia planning to minimize the risk of adverse events. It is important to emphasize that the risk of TdP rises significantly as various risk factors pile up. Preoperative risk assessment, treatment of correctible factors, and appropriate preventive measures will significantly decrease the incidence of life-threatening arrhythmias during the surgical procedure. Familiarity with potential complications, treatment options, and preventive measures are prerequisites for safe anesthesia management.

The QTc is most commonly measured using Bazett's formula (QTc = QT/RR^1/2^), or Fridericia formula (QTc = QT/RR^1/3^) (Drew et al., 2010). The latter one is preferentially used with higher heart rates. The upper limit of normal QTc is 470 msec in postpubertal males and 480 msec in postpubertal females (Berul, 2020). While measuring the QTc, one should keep in mind that there is a significant fluctuation of the interval during the day depending on the sympathetic activity and state of wakefulness (Brygger and Herrstedt, 2014). Values >480 msec on repeated testing or >460 msec in combination with a syncope will suggest LQTS (Staudt and Watkins, 2019). QTc >500 msec or an increase >60 msec is considered a significant risk factor for TdP and requires immediate intervention to avoid lethal arrhythmias (Brygger and Herrstedt, 2014). Additional findings in patients with LQTS include T-wave alterations, prolonged T_peak_-T_end_ interval, and polymorphic ventricular arrhythmias (Staudt and Watkins, 2019). In patients with LQTS, the EKG picture may be variable and the clinical manifestations may vary from absent to life-threatening arrhythmias such as sudden cardiac arrest (Brygger and Herrstedt, 2014). It is noteworthy to mention that up to 40% of patients with genetic predisposition do not demonstrate QT prolongation on a resting electrocardiogram (ECG). (Staudt and Watkins, 2019).

Patients diagnosed with QTc prolongation belong to two major groups: congenital, and acquired QTc prolongation (Curigliano et al., 2009; Staudt and Watkins, 2019). This distinction is, however, arbitrary. Even though it is convenient to think of QTc prolongation as occurring because of either congenital or acquired reasons, the phenomenon may sometimes involve a gene–environment interaction (Curigliano et al., 2009). Genetic mutations in patients with LQTS entail defects in structural proteins of ion channels involved in myocardial action potential generation, with resultant channelopathy and prolonged myocardial repolarization. The delayed repolarization puts patients at risk for sudden onset of ventricular tachyarrhythmias, most notably TdP (Staudt and Watkins, 2019).

The first genetic mutations implicated in development of hereditary QTc prolongation were described in mid-1990s, and currently around 1000 mutations in twelve distinct LQTS susceptibility genes have been identified that contribute to congenital predisposition to LQTS (Drew et al., 2010; Staudt and Watkins, 2019). Patients, who become symptomatic early in their life, are at higher risk for life-threatening events, including sudden cardiac death. The most common types of congenital LQTS are shown in Table 2. The syndrome is seen in one out of 2,000–3,000 people, and it may follow both autosomal dominant and recessive pattern with variable penetrance (Brygger and Herrstedt, 2014; Staudt and Watkins, 2019).

**TABLE 2:** Types of LQTS (Curigliano et al., 2009; Drew et al., 2010; Staudt and Watkins, 2019).

		Genetic mutation	Prophylaxis and therapy^a^	Comments
LQTS 1	75%	Loss of function mutations in KCNQ1 (slowly activating component in the delayed rectifier K^+^ current channel)	Beta blockers (very effective), MgSO_4_ IV, treat hypokalemia, hypocalcemia, implantable devices, thoracic sympathectomy	May be induced by sympathetic activation
LQTS 2		Loss of function mutations in KCNH2 (responsible for the rapidly activating component of the delayed rectifier K + current channel)	Beta blockers, MgSO_4_ IV (30 mg/kg), treat hypokalemia, hypocalcemia, implantable devices, thoracic sympathectomy	May be induced by sympathetic activation
LQTS 3		Gain of function mutations in SCN5A (responsible for encoding an inward sodium current channel)	Beta blockers (less effective), MgSO_4_ IV, treat hypokalemia, hypocalcemia, avoid bradycardia	May be induced by sleep or rest with slowing of heart rate
Less common forms of LQTS	5%	ANKB, ANK2, mink, IsK, KCNE1, MiRP1, KCNE2, Kir2, KCNJ2, CACNA1C	Similar to LQTS 1 and 2	
Genetically silent congenital LQTS	20%	No mutations revealed	Similar to LQTS 1 and 2	

LQTS, long QT syndrome.

^a^ defibrillation, cardioversion or pacing should be used in cases when conservative measures are ineffective, and the patient develops cardiovascular instability.

The congenital forms of the disease mostly remain silent, but in 10–12% of patients they initially present as sudden cardiac death. When left untreated, the mortality rate will reach 21% within a year after the initial syncope (Staudt and Watkins, 2019). The morbidity and mortality rates, however, significantly improve with appropriate therapy including beta blockers, correction of electrolyte deficiencies, and implantation of cardioverter-defibrillators, when indicated.

Besides genetic factors, many physiological conditions, disease states, and medications may also predispose to long QTc (Drew et al., 2010). These factors include:

✓ hypomagnesemia,
✓ hypokalemia,
✓ hypocalcemia,
✓ bradycardia,
✓ sleep,
✓ drugs reducing the activity of hepatic enzymes and the rate of drug degradation
✓ female sex,
✓ older age,
✓ ischemic heart disease,
✓ ventricular hypertrophy.

Presence of these factors and concomitant use of drugs with QTc prolonging potential may predispose to acquired QTc prolongation and trigger arrhythmias in genetically susceptible patients. Preoperative correction of modifiable factors (items one to six in the list) is a mandatory requirement in planning anesthesia, which will significantly reduce the risk of severe arrhythmias during surgery.

When preparing for general anesthesia, it is important to review the patient's medication list to identify drugs with potential QTc prolonging property (Curigliano et al., 2009; Berul, 2020). A short list of such drugs include:

✓ class Ia and III antiarrhythmics,
✓ macrolide antibiotics,
✓ sulfamethoxazole-trimethoprim,
✓ milrinone,
✓ vasopressin,
✓ tricyclic antidepressants,
✓ phenothiazines,
✓ many chemotherapeutic agents,
✓ several cholinergic antagonists,
✓ diuretics.

In clinical setting, it is difficult to assess the incidence and the extent of QTc prolongation caused by a specific drug, since most patients receive multiple drugs with QTc prolonging potential. Their cumulative effect is more pronounced and, in general, stronger effects are seen with higher rates of infusion and at maximal plasma concentrations. In patients with long QTc, the lowest effective doses of the drugs should be used and administered as slow infusions rather than bolus injections. Such an approach will reduce the risk of severe arrhythmias.

Aside from various medications used to treat patients in the perioperative period, there are drugs routinely used as part of anesthesia, which may also prolong the QTc: succinylcholine, isoflurane, sevoflurane, and desflurane (Staudt and Watkins, 2019). These agents, whenever possible, should be avoided or used in reduced doses in high risk patients.

## Relevance of QTc Prolongation to PONV Management

PONV is a distressing symptom complex, commonly encountered in the postoperative period. The incidence of vomiting may reach 30%, while the incidence of nausea is around 50% among general surgical patients and up to 80% in high-risk groups (Gan et al., 2020).

Several scoring systems have been developed to assess the risk of PONV, and among them the Apfel score is the most popular one. It considers 4 parameters, which positively correlate with the risk of PONV:

1 Female gender,
2 Non-smoking status,
3 History of PONV,
4 Use of postoperative opioid analgesics.

Presence of each of the parameters adds roughly 20% to the risk of PONV.

Numerous drugs with antiemetic properties have been successfully used to prevent and treat PONV (Table 1). Unfortunately, many of these drugs and their pharmacological groups share the potential for QTc prolongation. Patients with acquired or congenital forms of QTc prolongation present a group of increased risk for development of serious arrhythmias during surgery. In these patients, safe anesthesia management with PONV prophylaxis and treatment requires understanding of the underlying mechanisms of QTc prolongation and careful risk - benefit assessment. For most of those patients, antiemetic therapy may still be safely used, despite the potential for a transient QTc prolongation, since the EKG changes are mostly self-limited and remain clinically silent. However, all patients will require preoperative correction of predisposing correctible factors, including electrolyte disorders, preferential use of antiemetic medications devoid of QTc prolonging properties, and preparedness to intervene should serious arrhythmias take place. Appropriate medications, defibrillation/cardioversion and pacing equipment should be readily available.

The following approach may help to safely provide anesthesia in patients with a long QTc.

### Preoperative Period

Detailed family history of arrhythmias, episodes of syncope, and sudden death should be obtained. As part of preoperative assessment, all the existing modifiable risk factors should be revealed and corrected prior to surgery, whenever possible. This will include:

✓ correction of electrolyte disorders,
✓ stabilization of cardiovascular function,
✓ a review of the medication list with exclusion, if possible, of drugs with QTc prolonging properties,
✓ establishment of an anesthesia plan which will include use of safe anesthetics, adequate cardiovascular monitoring, ensure availability of drugs and equipment to treat intraoperative arrhythmias,
✓ PONV prophylaxis with drugs and non-pharmacological methods with no arrhythmogenic potential.

Patients with long QTc, who are at low risk for PONV, probably, do not need any prophylactic antiemetic premedication to avoid unnecessary risk for rare but well-described side effects (Gan et al., 2020).

Patients with increased PONV risk may safely receive midazolam, gabapentin, amisulpride or a scopolamine patch, whenever appropriate. Palonosetron may also be used before surgery, since no cases of QTc prolongation have been reported with the drug (Gan et al., 2020). However, there are no studies done on QTc at the time of the drug's C_max_. The NK_1_ receptor antagonist aprepitant and its prodrug fosaprepitant (available for IV infusion) can safely be used in patients with long QTc (Marbury et al., 2009). Alternatively, preoperative acupuncture at the P6 point can be recommended as a non-pharmacological and safe method of PONV prevention. Again, it is important to correct any preexisting electrolyte deficiencies (hypomagnesemia, hypokalemia, hypocalcemia) before anesthesia. In case of time restrictions, corrective therapy should continue during surgery.

### Intraoperative Period

Defibrillation/cardioversion and pacing equipment should be readily available during surgery. In patients with high risk for TdP and other serious arrhythmias, vapor anesthetics should be avoided or used with great caution. Patients with LQTS type 2 are more susceptible to vapor anesthetics, which may trigger serious arrhythmias in this group (Staudt and Watkins, 2019).

Total intravenous anesthesia with propofol and low dose opioids may be used safely. Alternatively, nitrous oxide and oxygen mixture with a low dose propofol (20–50 mcg/kg/min) infusion may be used in some patients, since nitrous oxide has minimal impact on PONV in low risk patients (Staudt and Watkins, 2019; Gan et al., 2020).

Dexamethasone can safely be used in all patients since it lacks any QTc prolonging properties. Dropridol, haloperidol, ondansetron and other drugs, with a potential for QTc prolongation, should be excluded in patients with QTc >480 mces. If QTc <480 msec and there is no history of arrhythmias or syncopes, droperidol (1.0–1.25 mg), or ondansetron (4–8 mg), may be used cautiously if diluted and injected slowly, since the risk of cardiac side effects of the drugs increases proportionally with the C_max_.

There has been a long debate about droperidol related to the black box warning generated by the FDA regarding the risk for TdP (Kantor, 2002; Habib and Gan, 2003; Kao et al., 2003; Shafer, 2004; Charbit et al., 2005; Charbit et al., 2008; Halloran and Barash, 2010; Perkins et al., 2015; Tracz and Owczuk, 2015; Lai and Huang, 2018). It is well known that butyrophenones, including droperidol and haloperidol, increase the QTc, and in some patients such a transient prolongation may trigger TdP or other arrhythmias. However, the extent of QTc prolongation caused by droperidol, like many other drugs, is transient and dose-dependent. Charbit. et al. (2008) compared the maximal placebo time-matched and baseline-subtracted QTc prolongation for droperidol and ondansetron in 16 patients and found a small but statistically significant difference between the drugs: the QTc prolongation was 25 ± 8 msec for droperidol and 17 ± 10 m sec for ondansetron (Charbit et al., 2008). Interestingly, Charbit B. and coauthors published the results of another study in 2005, where 85 patients with PONV were included (Charbit et al., 2005). In that study, the authors described a mean maximal QTc interval prolongation of 17 ± 9 m sec after droperidol occurring at the second minute and 20 ± 13 msec after ondansetron at the third minute. The authors concluded that when used in treatment of postoperative nausea and vomiting, a situation where prolongation of the QTc interval seems to occur, the safety of 5-hydroxytryptamine type 3 antagonists may not be superior to that of low-dose droperidol (Charbit et al., 2005). The discrepancies in these papers as well as many others can be explained by differences in study designs, patient selection criteria and differences in drug doses studied. In the latter study, the authors used 0.75 mg of intravenous droperidol vs. 1 mg in the previous study. This indicates that lower doses of droperidol are safer for use, and their cardiac effects are minimal. According to the American Academy of Emergency Medicine position statement published in 2015, in the year 2000, over 25 million unit doses of droperidol were sold and only 10 adverse cardiac events were related to doses of 1.25 mg or less as cited in (Perkins et al., 2015). All 10 of patients had confounding factors that could have explained the cardiac event.

Currently, the IV Consensus Guidelines for the Management of Postoperative Nausea and Vomiting recommend intravenous droperidol (0.625 mg) for PONV management (Gan et al., 2020).

As a precaution, or as a first line therapy, MgSO_4_ (30 mg/kg), and beta blockers, should be used if an arrhythmia is observed. MgSO_4_ is indicated even in patients with normal serum magnesium levels. If not corrected in the preoperative period, hypokalemia and hypocalcemia should be treated promptly. Depending on specific clinical scenarios, additional antiarrhythmic drugs and cardioversion may be required to control the intraoperative episodes of arrhythmia.

### Postoperative Period

If antiemetic treatment is required in the postoperative period, diluted slow injections of ondansetron, droperidol or haloperidol may be used in low risk patients. Small boluses of propofol may be used in high risk patients. Drugs with no QTc prolonging properties described in Table 1 may be used as well.

It is important to minimize opioid administration to further reduce the risk of PONV, and non-opioid analgesics should be considered for pain control. EKG monitoring should be continued in high risk patients in the postoperative period.

## Conclusion

Proper planning and preparation to treat adverse events, use of drugs with no or minimal QTc prolonging properties in the perioperative period can minimize the risks of long QTc and avoid life threatening arrhythmias. Additional studies and clinical trials will help to develop specific strategies to manage the perioperative nausea and vomiting is this group of high risk.

## Author Contributions

SS suggested the topic as a new one and deserving clarification, since there are not enough publications in the field. He reviewed the literature sources, wrote the manuscript and prepared the tables. He serves as the corresponding author for the submission. NS ran literature search and sorting. She participated in writing the manuscript and preparing the tables. WA ran literature search, participated in writing the manuscript and preparation of tables. SB supervised the process and distributed the roles. She participated in literature search, writing of the manuscript, and preparation of the tables. She made final editions as a cardiac anesthesiologist.

## Conflict of Interest

The authors declare that the research was conducted in the absence of any commercial or financial relationships that could be construed as a potential conflict of interest.

The reviewer BR declared a shared affiliation with the authors to the handling editor at time of review.
